# Milk and Milk-Contracts
*Previous articles appeared on May 3, 10, and 17.


**Published:** 1913-05-24

**Authors:** Everitt E. Norton

**Affiliations:** Medical Superintendent of Isleworth Infirmary.


### MILK AND MILK-CONTRACTS.*

By EVERITT E. NORTON, M.D., D.P.H., etc., Medical Superintendent of Isleworth Infirmary.

### IV.—Milk Dangers.

In attempting to criticise the conditions which I have briefly described as those under which milk is now largely supplied, it may be well to state the inherent dangers and difficulties which have to be contended with. It is clear that these difficulties, and the need for the adoption of means to overcome them, are fully recognised to have resulted almost entirely from conditions which have gradually (and in some degree necessarily) arisen in connection with the milk supply of towns, and especially of such an aggregation of towns as modern London. They are:—

(i) Those depending upon the animal source, fluidity, and characters of milk: (*a*) Its liability to undergo a specific form of decomposition, consequent upon the growth of organisms of certain types. (*b*) The frequency of its infection by other organisms, some of them possibly pathogenic to man, and its remarkable suitability as a culture medium for the growth of such organisms.

(ii) Those depending upon the immense distances from which it is necessary to bring supplies and the long time occupied in transit.

(iii) The (added and enhancing) effects of the defective methods employed in collecting, conveying, and handling of milk.

### Defects in the Process of Milking.

It must be accepted as a fact that the collection from cows, or from any animals, of milk sterile of bacteria is an ideal beyond hope of fulfilment. Though it be granted that some portion of the milk as it leaves the udder may be sterile, no method of collecting it (even by experimental mechanical milkers, which have given disappointing results, in use) in a sterile condition has proved, or is likely to prove, practicable. Commercially, certainly, there can be no milk in a state even nearly approaching sterility. Dr. F. J. H. Coutts finds even tinned condensed milk to be, on examination, capable of yielding growths. Further, the ordinary farm conditions tend only too often to render more certain the introduction of a variety of forms of dirt with the accompanying organisms; pathogenic infections, derived either from disease of the cow or from other sources, amongst the number. The precise numerical bacterial content of milk at the farm, which is undoubtedly very variable, is of less consequence than the fact that a bacterial content exists and that it is capable of limitation by the enforcement of cleanliness as regards the cows, milkers, cowsheds, and vessels. Dr. Thomas Orr speaks of many "extraordinary instances of carelessness and ignorance in a trade concerned with the supply of an important foodstuff," and similar instances have been observed by every competent student of the subject of our milk supply.

It is impossible to lay too great stress upon the importance of cleanliness in farm methods. It is at the farm that the greatest amount of preventable contamination occurs. It is at the farm, and at the farm alone, that it is possible to secure the protection of milk from most of the filthy and dangerous pollutions to which it has become so lamentably liable; and upon the success of the efforts made at the farm depends the value and safety of the supply. That reform in this direction is not easily to be carried out is certain. The attack upon custom, self-interest, laziness, and ignorance is one which cannot be effectively sustained by private effort alone, but gross pollution is preventable and should be prevented.

It is impracticable for the purchasers, in London, of milk under contract to undertake themselves the supervision of farm conditions and methods. It is possible, and it is necessary, for them to see that their interests are adequately protected by qualified representatives appointed by them. Milk procured under more stringent restrictions will inevitably cost more, for a time at least; it has been estimated that the grooming and washing of cows adds alone a farthing a gallon to the cost of production.

The fact that all milks are prone to contain a sediment of particles, visible to the naked eye and largely removable by straining, is apparent to anyone who has seen an efficient strainer in use; and milk deposits a "slime" which may be collected after separation by means of a centrifugal machine. Sediment is dirt which gains access to the milk at the cowshed. Leucocytes and cellular debris (from the teats and ducts), vegetable particles from manure, granular dust particles, hairs, fragments of straw, yeasts, and bacteria are mentioned by Orr as being commonly found.

The necessary limitations of such an article as this prevent me from dealing at any length with the bacteriology of milk, nor is it necessary for my purpose that I should attempt to do so. Milk, as we know it, is and must be a fluid teeming with bacteria of certain types; it is of the greatest importance that the essentially morbific bacteria should be excluded. London milk may contain a greater numerical proportion of bacteria than sewage, and the one scientific test, by which it is at present possible and practicable to judge and standardise milk supplies, is that of bacterial content.

### American Methods.

The method may appear at first sight to be a troublesome and costly one, but the difficulty of expense has been surmounted by progressive milk-dealers in America, who now habitually make use of it as a part of their routine. American local authorities have established, and have made efforts to enforce, numerical bacteria standards: in Chicago, for example, milk arriving in the city must not contain more than 1,000,000 bacteria per

* Previous articles appeared on May 3, 10, and 17.

cubic centimetre during the months of May to September inclusive, and not over 500,000 during the other months of the year. Whilst the difficulties of enforcing such standards are acknowledged, it is evident that a high bacterial content is an indication of the greatest value that milk is probably "dirty, old, or warm."

The results of very numerous examinations conclusively prove with what extraordinary rapidity bacterial development takes place when milk is kept for a long time at a moderately warm temperature. It is, of course, in summer that a high bacterial count is more frequently recorded. Dr. Ralph Vincent quotes the following observations, which were made at the laboratory of the New York Health Department:—

A sample of milk taken under good conditions contained, immediately after milking, in each drop 300 bacteria. It was cooled to 45° F. and kept at this temperature. After twenty-four hours it contained in each drop 200 bacteria, after forty-eight hours 900 bacteria, and after seventy-two hours 150,000 bacteria.

Another sample, taken in a dirty barn, cooled, and kept at 52° F., contained at first in each drop 2,000 bacteria; after twenty-four hours it contained in each drop 6,000 bacteria, after forty-eight hours 245,000 bacteria, and after seventy-two hours 16,500,000 bacteria.

Freudenreich (quoted by Orr) gives very interesting ratios of the increase of bacteria at different temperatures and at different times.

Sir George Newman found, in his investigation of the milk supply of Finsbury, that the average number of bacteria in unpreserved milk was 2,370,000 per cubic centimetre; pus and dirt were frequently found. Eastes examined 186 samples of milk, and found "pus or muco-pus" in 134 cases, blood in 24, and streptococci in 106; he considered 80 per cent. of the milk to be unfit for human consumption.

### The Source of Infection.

Sporadic cases of diarrhœa have their origin in contamination by infective dust (Newsholme), and Dr. Orr found that the glucose-fermenting or "intestinal" organisms, streptococci, and bacilli enteritidis sporogenes (Klein) are introduced with by far the greatest frequency at the farm.

The differentiation and pathological significance of leucocytes and pus-cells demand further investigation.

When such specific infective diseases as scarlatina, diphtheria, or enteric fever are disseminated by milk, infection is usually conveyed to it either by the milkers or other persons about the farm, or by the water used at the farm. Such infections may arise, and frequently have arisen, "from the grossest neglect of the most elementary precautions" (Vincent).

The uncertainty of the results of examination for the bacilli of tubercle in milk is well known, and only renders practical precautions to prevent infection by tubercle the more necessary. Dr. Newsholme (now Medical Officer to the Local Government Board) says that when in milch-cows "there is tuberculosis of the udder the milk is found to be dangerously infectious." He quotes the results of Dr. Sidney Martin's experiments and the Report of the Royal Commission on Tuberculosis that, "according to our experience, the condition required for ensuring to the milk of tuberculous cows the ability to produce tuberculosis in the consumers of their milk is *tuberculous disease of the cow affecting the udder.* It should be noted that this affection of the udder is not peculiar to tuberculosis in an advanced stage, but may be found also in mild cases." Further, "the milk of cows with tuberculosis of the udder possesses a virulence which can only be described as extraordinary." Sir J. MacFadyean has estimated that some 2 per cent. of the cows in the milking herds of this country have tuberculous disease of the udder, whilst Professor Delépine puts the proportion at 3.7 per cent. The importance of the subject is shown by the opinion (quoted by Dr. Newsholme from the Report of the Royal Commission) that "the largest part of the tuberculosis which man obtains through his food is by means of milk containing tuberculous matter." "Tuberculosis of the udder can rarely be differentiated from other forms of udder disease by the ordinary stockowner or dairyman. . . ."

### A Suggested Remedy.

Whilst the powers for dealing with cases of this condition have been extended, some of them (says Dr. Newsholme) "remain a dead letter in most urban districts," and they are still incomplete. He adds: "Apart from the enforcement of public health regulations, public protection might be entirely secured under the ordinary conditions of commercial life if the public were willing to pay a little more . . . and it is not unlikely that the additional expenditure at first incurred by the enterprising large farmer . . . would eventually be recouped by the more permanent healthiness of his herd."

It has been proposed to exclude sediment (dirt) from milk by milking directly into a pail having an opening of small diameter protected by a strainer. Conn (quoted by Orr) says that a sterile cotton-wool strainer covering an opening of about six inches diameter in such a pail should catch 66 per cent. of the dirt. The New York Milk Commission's instructions enjoin the use of milking-pails having an opening not exceeding eight inches in diameter. In the absence of any such arrangement, careful straining should invariably be practised immediately after the milk is removed from the cowshed. Particles of manure only too commonly find their way into the milk, especially in winter, and, of course, when the cows are not groomed or cleaned in any way; such particles are usually of old dried manure, and it is evident that the bacterial pollution caused by them, or by dust, etc., and in which lies the real danger of their presence, commences immediately, occurs rapidly, and increases as they become softened. The removal by straining of such particles after a considerable lapse of time is of comparatively little use.

